# An isoflavone derivative potently inhibits the angiogenesis and progression of triple-negative breast cancer by targeting the MTA2/SerRS/VEGFA pathway

**DOI:** 10.20892/j.issn.2095-3941.2020.0010

**Published:** 2020-08-15

**Authors:** Xiaotong Zhang, Gengyi Zou, Xiyang Li, Lun Wang, Tianyu Xie, Jin Zhao, Longlong Wang, Shunchang Jiao, Rong Xiang, Haoyu Ye, Yi Shi

**Affiliations:** ^1^School of Medicine, Nankai University, Tianjin 300071, China; ^2^2011 Project Collaborative Innovation Center for Biotherapy of Ministry of Education, Tianjin 300071, China; ^3^Department of Oncology, Chinese PLA General Hospital, Beijing 100853, China; ^4^State Key Laboratory of Biotherapy and Cancer Center, West China Hospital, Sichuan University, and Collaborative Innovation Center for Biotherapy, Chengdu 610041, China

**Keywords:** Isoflavone, MTA2, SerRS, tumor angiogenesis, VEGFA

## Abstract

**Objective:** Angiogenesis plays a vital role in tumor growth and metastasis. Here, we aimed to find novel efficient antiangiogenic molecules targeting vascular endothelial growth factor A (VEGFA ) at the transcriptional level to treat triple-negative breast cancer (TNBC).

**Methods:** We used a cell-based seryl tRNA synthetase (SerRS) promoter-driven dual-luciferase reporter system to screen an in-house library of 384 naturally occurring small molecules and their derivatives to find candidate molecules that could upregulate the expression of SerRS, a potent transcriptional repressor of VEGFA. The levels of SerRS and VEGFA were examined by quantitative RT-PCR (qRT-PCR), western blotting, and/or ELISAs in TNBC cells after candidate molecule administration. Zebrafish, the Matrigel plug angiogenesis assay in mice, the TNBC allograft, and xenograft mouse models were used to evaluate the *in vivo* anti-angiogenic and anti-cancer activities. Furthermore, the potential direct targets of the candidates were identified by proteomics and biochemical studies.

**Results:** We found the most active compound was 3-(4-methoxyphenyl) quinolin-4(1H)-one (MEQ), an isoflavone derivative. In TNBC cells, MEQ treatment resulted in increased SerRS mRNA (*P* < 0.001) and protein levels and downregulated VEGFA production. Both the vascular development of zebrafish and Matrigel plug angiogenesis in mice were inhibited by MEQ. MEQ also suppressed the angiogenesis in TNBC allografts and xenografts in mice, resulting in inhibited tumor growth and prolonged overall survival (*P* < 0.05). Finally, we found that MEQ regulated SerRS transcription by interacting with MTA2 (Metastasis Associated 1 Family Member 2).

**Conclusions:** Our findings suggested that the MTA2/SerRS/VEGFA axis is a drug-treatable anti-angiogenic target, and MEQ is a promising anti-tumor molecule that merits further investigation for clinical applications.

## Introduction

It is estimated that over 2 million women in the world are diagnosed with breast cancer every year^1^. Among these, at least 15% are classified as triple-negative^1^. Triple-negative breast cancer (TNBC) is characterized by younger age occurrence, higher rates of relapse, greater metastatic potential, and shorter overall survival compared with the hormone receptor-positive/human epidermal growth factor 2 receptor (HER2) negative (70% of patients) and HER2 positive (15%–20% of patients) phenotypes^2,3^. Based on the molecular pathogenesis of these two cancer subtypes, multiple effective targeted therapies have been developed, such as endocrine agents for estrogen receptor (ER)-positive or progesterone receptor (PR)-positive breast cancers, anti-HER2 antibodies (such as trastuzumab and pertuzumab), and small-molecule tyrosine kinase inhibitors (such as lapatinib and neratinib) for HER2-positive types^4^. However, TNBC lacks the expression of both the ER and PR and does not overexpress HER2^5^. And the specific molecular pathophysiology of TNBC remains poorly understood. Therefore, there is currently no effective, specifically targeted therapy available for TNBC^4^.

Targeting angiogenesis is a valuable strategy to inhibit solid tumor development and progression because solid tumors (like TNBC) cannot grow beyond a few millimeters in size or spread without a blood supply^6^. Tumor cells can induce the blood supply by secreting angiogenic factors, like VEGFA, to stimulate angiogenesis^7^. Bevacizumab, an anti-angiogenic inhibitor targeting VEGFA, is approved to treat several tumor types, including TNBC^8^. However, due to the relative poor tissue penetration of bevacizumab for its large size, the addition of bevacizumab to traditional chemotherapy drugs did not result in a statistically significant increase in the numeric rate of pathologic complete response (pCR)^9,10^. Also, the remaining and still active VEGFA after bevacizumab treatment may still be enough to induce angiogenesis. Several adverse effects of bevacizumab, such as headache, hypertension, proteinuria, taste alteration, and rectal hemorrhage have been reported^10,11^. Thus, novel small molecule drugs with good cell and tissue permeability, if able to effectively reduce the VEGFA level in tumor tissue and having few side effects, could be promising therapeutic agents for TNBC.

We previously reported that seryl tRNA synthetase (SerRS), which transfers L-serine to its specific tRNA for protein biosynthesis in the cytoplasm, was also a potent transcriptional repressor of VEGFA^12^. Furthermore, SerRS counteracts the effects of c-Myc and NFκB^12,13^ in modulating VEGFA expression. So, increasing SerRS expression could be a promising strategy to inhibit abnormal VEGFA expression and tumor angiogenesis. Although gene therapy is a method to directly introduce more SerRS protein in cells, the technology is still undeveloped and has potential risks for clinical applications^14,15^. Identifying small molecular drugs targeting SerRS expression may therefore be a more promising anti-angiogenesis strategy for the treatment of TNBC.

Natural small molecules isolated from traditional Chinese herbs are presently considered as optimal cancer drug candidates for their structural diversity, good cell permeability, low toxicity, and nonimmunogenic profiles^16^. Because of their evolutionary advantage, for high throughput screening, a library of natural small molecules is a better resource to discover molecules with fewer side effects than a synthetic compound library^17,18^. Derivatives based on natural small molecules also can possess improved pharmaceutical characteristics and minimal side effects. Based on these considerations, we established an in-house library consisting of 384 natural small molecules and their derivatives, which was screened with a TNBC cell-based and SerRS promoter-driven luciferase reporter system. We identified MEQ as a novel anti-angiogenesis agent showing robust activity to inhibit VEGFA and TNBC progression by targeting SerRS expression in both *in vitro* and *in vivo* model systems.

## Materials and methods

### Cell culture

MDA-MB-231 and 4T1 cells were purchased from the American Type Tissue Collection (Manassas, VA, USA). Both MDA-MB-231 cells and 4T1 cells were cultured in Dulbecco’s Modified Eagle’s Medium (Biological Industries, Cromwell, CT, USA) supplemented with 10% fetal bovine serum (Biological Industries) under 5% CO_2_ at 37 °C.

### Chemicals

Our in-house small molecule library (384 naturally occurring small molecules or their derivatives) was established by the State Key Laboratory of Biotherapy and Cancer Center, West China Hospital, Sichuan University, China. All compounds were dissolved in dimethyl sulfoxide (DMSO) and stored at −20 °C at a concentration of 20 mM.

### Dual-luciferase vector construction and the dual-luciferase reporter assay

The gene sequence of the SerRS promoter was amplified by the polymerase chain reaction (PCR) from the HEK 293 cell line using a forward primer: 5′-TCAGGTACC AGTAGAGACGGGATTTTGTCATG-3′ and reverse primer: 5′-TCACTCGAGCAAATCCAGATCCAGCACCATCTTC-3′ with Kpn I and Xho I restriction enzyme sites. The fragment was then subcloned into the pGL4.11 [luc2P] vector. A stably-transfected MDA-MB-231 cell line containing a SerRS promoter-driven firefly luciferase [pGL4.11 (luc2P)] and Renilla (pRL-SV40) was constructed for the purpose of screening for molecules that activated the promoter of SerRS. Stably-transfected MDA-MB-231 cells were seeded into 24-well plates, incubated for 24 h, exposed to experimental compounds or DMSO as a control for 48 h, and the luciferase activities measured (Promega, Madison, WI, USA).

### Drug toxicity assay

BALB/c mice (6–8 weeks of age) were conditioned for 1 week in our pathogen-free animal facility. BALB/c mice (6–8 weeks of age, *n* = 3) were treated with 100 mg/kg MEQ or DMSO (intraperitoneal injection, twice a day) and their weights measured for 4 weeks (once every 4 days) before sacrifice. Blood samples were taken by eyeball extirpation, and the hearts, livers, spleens, lungs, kidneys, and brains were harvested after sacrifice. Blood tests were performed with a hematology analyzer (Celltac E; Nihon Kohden, Tokyo, Japan).

### Immunoblotting

Cell or tissue lysates were prepared with lysis buffer [200 mM Tris-Cl (pH 7.5), 1.5 M NaCl, 20 mM EDTA, 10% Triton X-100, 10 mM Na_3_VO_4_·12 H_2_O, and 1% protease inhibitor cocktail (Sigma-Aldrich St. Louis, MO, USA)] on ice. Protein concentrations of samples were determined using a Pierce™ BCA Protein Assay Kit (Thermo Fisher Scientific, Waltham, MA, USA). Primary antibodies were as follows: anti-SerRS (1:1,000; made in the laboratory), anti-VEGFA (1:1,000; Santa Cruz Biotechnology, Santa Cruz, CA, USA), anti-β-actin (1:5,000; Santa Cruz Biotechnology), and anti-MTA2 (1:1,000, Santa Cruz Biotechnology). All antibodies recognized both human and mouse proteins. After SDS-PAGE and western blotting, the bands were detected by chemiluminescence using an ECL kit (Thermo Fisher Scientific).

### Quantitative real-time polymerase chain reaction (qRT-PCR)

Total RNA was extracted from the indicated cells, zebrafish tissues, or mouse tumors using TRIzol reagent (Yeasen Biotechnology, Shanghai, China). One μg of total RNA from each sample was reverse transcribed into cDNA using M-MLV Reverse Transcriptase (Promega). All real-time qPCRs were performed using a LightCycler 96 (Roche, Basel, Switzerland) Real-Time PCR system with Hieff qPCR SYBR Green Master Mix (Yeasen Biotech). The following primers were used for the qPCR reactions: 5′-AAGAAAGCAGCAGCAAGAGACG-3′ and 5′-CATGCGAGGAGACAGGAACATC-3′ for human *SARS1*; 5′-GAGGGCAGAATCATCACGAAG-3′ and 5′-TGTGCTGT AGGAAGCTCATCTCTC-3′ for human *VEGFA*; 5′-CGTCA CCAACTGGGACGA-3′ and 5′-ATGGGGGAGGGCATACC-3′ for human *ACTB*; 5′-TGCGACCACTCGTGTCATCT-3′ and 5′-GAGACTGTGGAGGGCGTGTC-3′ for zebrafish *sars1*; 5′-GGCTCTCCTCCATCTGTCTGC-3′ and 5′-CAGTGGTT TTCTTTCTTTGCTTTG-3′ for zebrafish *vegfa*; 5′-TCACCACC ACAGCCGAAAGAG-3′ and 5′-GTCAGCAATGCCAGGGTA CAT-3′ for zebrafish *actb1*; 5′-ACCTGGTGGTGATGGTAG ATGG-3′ and 5′-AAGGGGGTGTAGATTGGAGTGT-3′ for mouse *Sars*; 5′-GTCCGATTGAGACCCTGGTG-3′ and 5′-TTG ACCCTTTCCCTTTCCTCG-3′ for mouse *Vegfa*; and 5′-GGCT GTATTCCCCTCCATCG-3′ and 5′-GCACAGGGTGCTCCT CAG-3′ for mouse *Actb*. The PCR reaction program was the following: 95 °C for 10 min, followed by 45 cycles of 95 °C for 20 s, and 60 °C for 1 min, ending with 95 °C for 2 min. All experiments were carried out in triplicate. The results are expressed as 2-ΔΔCT values and the SEM, and were in triplicate. The mRNA levels of SerRS and VEGFA were normalized to that of β-actin. Student’s *t*-test was used to analyze the difference between two groups.

### Enzyme-linked immunosorbent assay (ELISA)

Supernatants from MDA-MB-231 cells incubated with MEQ or DMSO for 48 h were collected. For the detection and quantification of secreted VEGFA, an ELISA was used according to the manufacturer’s instructions with signal detected at 450 nm (DLDEVELOP, Wuxi, China). Total protein was measured for normalization using a Pierce™ BCA Protein Assay Kit (Thermo Fisher Scientific/Pierce).

### Zebrafish angiogenesis model

Tg (Fli1a: EGFP) transgenic zebrafish were raised in a constant temperature (28.5 °C) room with a 14:10 h day/night cycle. Male and female zebrafish were randomly selected for copulation with a ratio of 3:2 and separated for 12 h before mating. Dead or unfertilized embryos were removed and those in good condition were kept in E3 embryo medium (5 mmol/L NaCl, 0.17 mmol/L KCl, 0.33 mmol/L CaCl_2_, and 0.33 mmol/L MgSO_4_, pH 7.2) at 28.5 °C. E3 embryo medium was changed to E3 plus 0.003% 1-phenyl-2-thiourea at 12 h post-fertilization. At the same time, the embryos were transferred to 24-well plates with 10 embryos per well and incubated with 10 μM MEQ or 0.1% DMSO. After 72 h of treatment, images of larval intersegmental vessels were captured using a DP72 digital camera mounted on an SZX16 dissecting microscope (Olympus, Tokyo, Japan).

### Matrigel plug assay

Matrigel (BD Biosciences, San Jose, CA, USA) was extracted from Engelbreth-Holm-Swarm mouse sarcomas. It consisted of extracellular matrix components and growth factors. Matrigel was stored at −20 °C and thawed at 4 °C before use. A total of 210 μL of a mixture of Matrigel (Matrigel:DMEM = 1:2), 1 × 10^6^ 4T1 cells, and 0.2 μL MEQ (10 mM) or 0.2 μL DMSO were injected subcutaneously into BALB/c mice. After 7 days, the Matrigel plug was removed and new blood vessel formation in the Matrigel plug was analyzed by immunofluorescence for CD31 and immunohistochemical (IHC) staining for SerRS and VEGFA.

### Animal studies

All mice were evaluated and noted to be healthy and free of disease before tumor implantation. Female BALB/c mice (6 weeks) and BALB/c NOD-SCID mice (6 weeks) were from SPF Biotechnology (Beijing, China). All animal experiments were approved by the Animal Care and Use Committee at Nankai University (Tianjin, China). After 1 week acclimation in the pathogen-free animal facility, 1 × 10^5^ 4T1 cells and 1 × 10^5^ MDA-MB-231 cells were injected into the #2 mammary fat pads of BALB/c mice and NOD-SCID mice, respectively. Mice were randomly divided into three groups (BALB/c mice) or two groups (NOD-SCID mice), and 1 week after the tumor cells injection, the control group received DMSO (the same volume as MEQ), while the other groups received MEQ at either 100 mg/kg or 200 mg/kg by intraperitoneal injection every other day. The volume (V) of each tumor was measured every 4 days and calculated using the formula: V = length × width^2^/2. Tumors were excised for analysis at the end of the expriments.

### Immunofluorescence (IF), hematoxylin and eosin (H&E), and IHC staining

Paraffin sections of Matrigel plugs were stained with a primary antibody against CD31 (1:50; Abcam, Cambridge, UK), goat-anti-rabbit Alexa 594 secondary antibody (ZSGB-Biotech, Beijing, China), and 4′,6-diamidino-2-phenylindole (DAPI) (Sigma-Aldrich) for nuclear staining and the IF was assessed. Sections were photographed using an FV-1000 laser scanning confocal biological microscope (Olympus). H&E staining of paraffin-embedded tumor sections from both mouse TNBC models and organs from the drug toxicity test mouse model was conducted for overall morphological observations. Tumors were also analyzed using SerRS (1:500; antiserum made in the laboratory), VEGFA (1:50; Santa Cruz Biotechnology), CD31 (1:50; Abcam), Ki67 (1:200; Abcam), and cleaved caspase-3 (CC3, 1:200; Cell Signaling Technology, Danvers, MA, USA) primary antibodies for immunohistochemical analyses. The DAPI was used for labeling the nucleus. Numbers of immune positive cells were quantified using ImageJ software, version 1.50 (National Institutes of Health, Bethesda, MD, USA). All images used the same exposure conditions.

### The shRNAs

DNAs expressing a short-hairpin RNA (shRNA) designed against the human *SerRS* (5′-AAAAGGCATAGGGACCCATC ATTGATTGGATCCAATCAATGATGGGTCCCTATG CC-3′) and human *MTA2* (5′-AAAAGCAGATCGACCAGTT TCTTGTTTGGATCCAAACAAGAAACTGGTCGAT CTGC-3′) genes were inserted into the pLV-H1-ef1α-puro plasmid.

### Streptavidin-biotin affinity pull-down assay

MDA-MB-231 cells were harvested, and lysates prepared and incubated with free biotin (MedChemExpress, Monmouth Junction, NJ, USA) or MEQ-biotin (10 μM) with rotation for 5 h at 4 °C. Recombinant streptavidin agarose beads were then added and incubated with rotation at 4 °C overnight. The beads were washed three times and bead-bound proteins separated by SDS-PAGE. Protein bands were visualized by silver staining. Bands between 70 kDa and 120 kDa were cut out for mass spectrometric analyses. MTA2 was detected using western blotting of the same streptavidin agarose bead-binding proteins.

### Statistical analysis

Data are presented as the mean ± SEM. Statistical analysis was performed using an unpaired Student’s* t-*test for comparisons of two groups. Analysis of variance (ANOVA) was used for multiple comparisons. *P* < 0.05, *P* < 0.01, or *P* < 0.001 were considered statistically significant and indicated by *, **, or ***, respectively. All statistics were performed using Prism 5 software (GraphPad, San Diego, CA, USA).

## Results

### Screening the natural small molecule library for compounds that activated the promoter of SerRS

**Figure 1:**
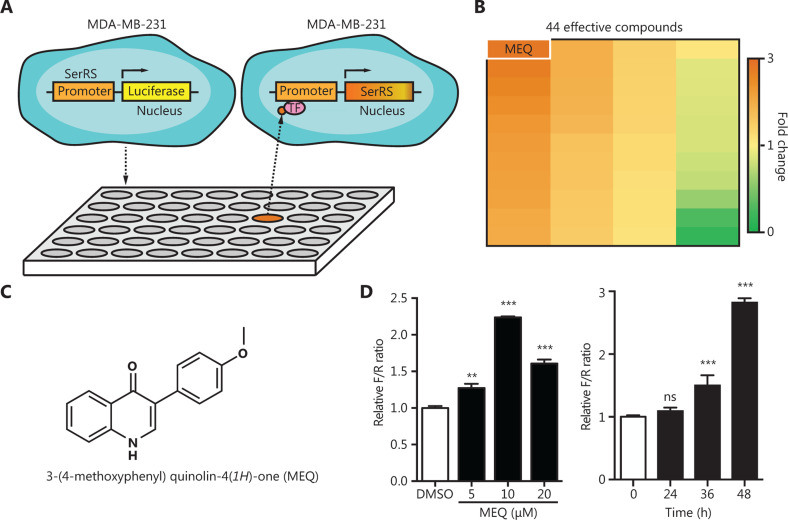
High throughput screening for compounds that act on the SerRS promoter. (A) Scheme of the dual luciferase reporter-based high throughput screening. (B) Heat map of the luciferase/Renilla ratios for 44 compounds responsive to the SerRS promoter. (C) The molecular structure of MEQ. (D) Relative firefly/Renilla (F/R) ratios for MEQ at indicated dosages and time points. Data are plotted as means ± SEM (*n* = 3; ns, not significant; **P < 0.01; ***P < 0.001 using Student’s *t*-test.

High-throughput screening has been developed as an efficient method for drug discovery. We used the TNBC cell line, MDA-MB-231, which was stably-transfected with a SerRS promoter-driven firefly luciferase gene and the Renilla luciferase gene, to screen a small molecule library containing 384 naturally occurring small molecules and their derivatives from our in-house drug library, each at a concentration of 10 μM (**Figure 1A**). At 48 h post-incubation, we used the firefly/Renilla ratio to determine the effect of these molecules on the SerRS promoter. Compared with the control group treated with DMSO, 34 compounds showed activation of the SerRS promoter, while 10 compounds showed the opposite activity (**Figure 1B**). Among the 34 small molecules that activated the SerRS promoter, MEQ, an isoflavone derivative synthesized by the method reported by Santosh Rajput^19^, exhibited the strongest activity (**Figure 1B and 1C**). MEQ activated the SerRS promoter at less than 10 μM without cellular toxicity and reached maximum activation at 48 h in MDA-MB-231 cells (*P* < 0.001) (**Figure 1D**).

### MEQ inhibited VEGFA expression by upregulating SerRS transcription in MDA-MB-231 cells

**Figure 2:**
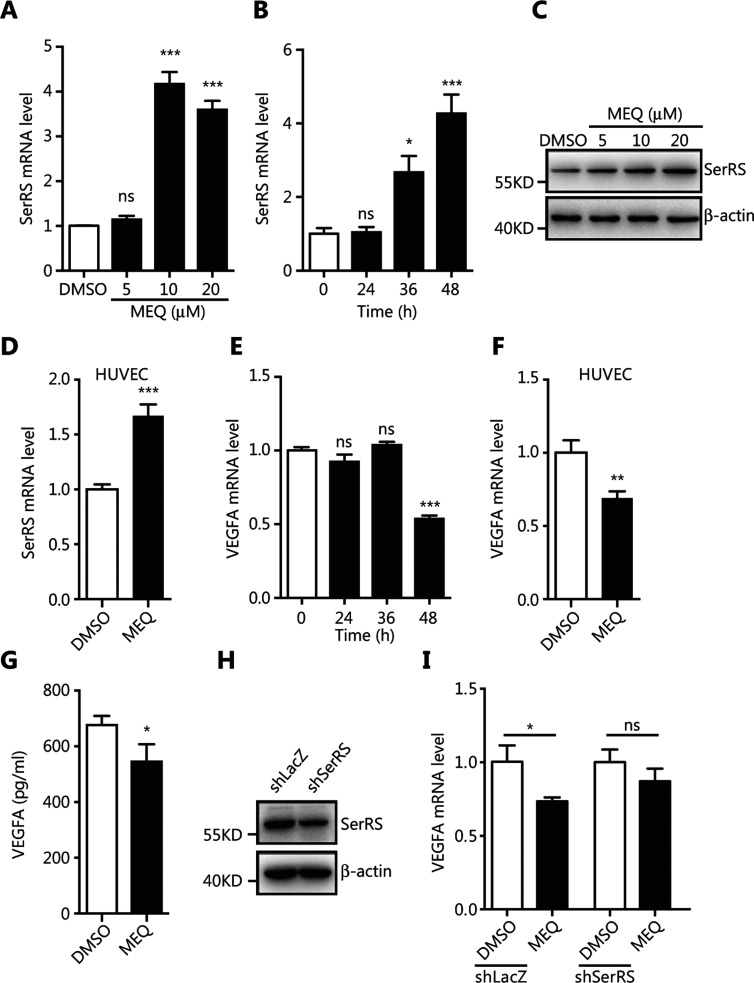
MEQ inhibits VEGFA expression by upregulating SerRS in cultured cells. (A, B) The qRT-PCR shows SerRS expression in MDA-MB-231 cells treated with MEQ at indicated dosages or with dimethyl sulfoxide (DMSO) as a vehicle control for 48 h (A) or treated with 10 μM MEQ for the indicated time periods (B). (C) Western blotting to show SerRS protein levels in MDA-MB-231 cells treated with the indicated dosages of MEQ or DMSO for 48 h. (D) The qRT-PCR shows SerRS expression in human umbilical vein endothelial cells (HUVECs) cells treated with 10 μM of MEQ for 48 h. (E, F) The qRT-PCR shows vascular endothelial growth factor A (VEGFA) expression in MDA-MB-231 cells treated with MEQ for the indicated time periods (E) and in HUVEC cells treated with 10 μM of MEQ for 48 h (F). (G) ELISA analyses of secreted VEGFA from MDA-MB-231 cells treated with 10 μM MEQ for 48 h. (H) Western blotting shows the knockdown efficiency of shRNA against SerRS (shSerRS) with a shRNA against* lacZ* gene (shLacZ) as a negative control. (I) The qRT-PCR shows VEGFA expression in shSerRS or shLacZ transfected MDA-MB-231 cells treated with 10 μM MEQ for 48 h. All bar graphs are plotted as the mean ± SEM (*n* = 3; ns, not significant; *P < 0.05; **P < 0.01; ***P < 0.001 using Student’s *t*-test.

To verify that MEQ upregulated the expression of endogenous SerRS, we treated MDA-MB-231 cells with MEQ at different dosages and for different time periods before SerRS expression was examined by qRT-PCR and western blotting. Consistent with the results from the reporter system, MEQ upregulated the expression of SerRS at 10 μM within 48 h (*P* < 0.001) (**Figure 2A–2C**). In addition, MEQ also enhanced SerRS expression in human umbilical vein endothelial cells (HUVEC) (*P* < 0.001) (**Figure 2D**). Given that SerRS is a potent transcriptional repressor of VEGFA^12,13^, we further examined the mRNA and protein levels of VEGFA in MEQ-treated MDA-MB-231 and HUVEC cells. **Figure 2E** shows that 10 M of MEQ reduced VEGFA mRNA by 50% within 48 h in MDA-MB-231 cells (*P* < 0.001), similar to the results in HUVEC cells (*P* < 0.01) (**Figure 2F**). Consistently, MEQ treatment also reduced secreted VEGFA (*P* < 0.05) (**Figure 2G**).

To further investigate if the MEQ-induced decrease in VEGFA expression was through its regulation of SerRS, we knocked down SerRS in MDA-MB-231 cells by shRNA (**Figure 2H**). After the knockdown, MEQ could no longer inhibit VEGFA expression in SerRS-silenced cells (**Figure 2I**), confirming that MEQ suppressed VEGFA expression *via* its regulation of SerRS.

### MEQ inhibited angiogenesis in vivo

**Figure 3:**
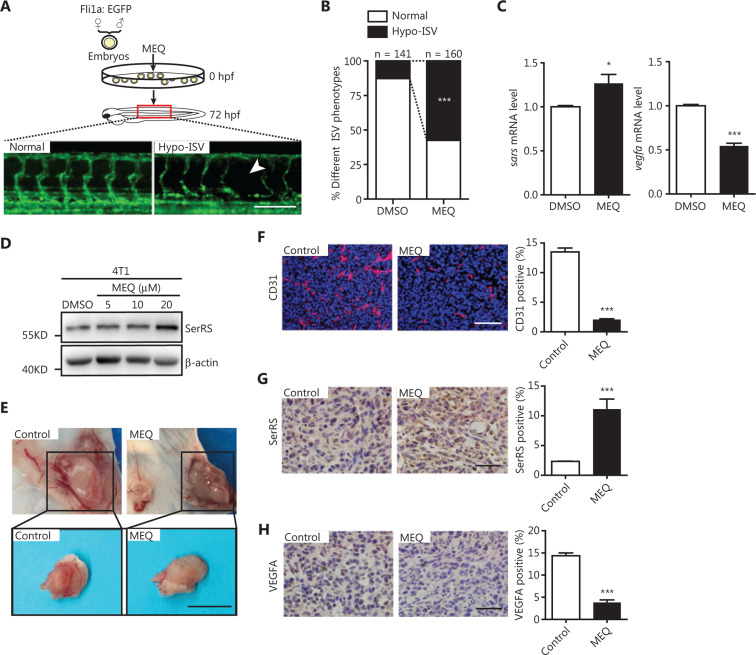
MEQ inhibits angiogenesis *in vivo*. (A, B) The embryos of transgenic zebrafish (Tg [Fli1a: EGFP]) at 1-2 cell stage were injected with MEQ and vascular development was monitored at 72 h post-fertilization (A) and the percentage of fish with deficiency in intersegmental vessels (Hypo-ISV) were analyzed (B). ***P < 0.001 using the χ^2^-test. (C) The qRT-PCR results show the expression of *Sars* (i.e., fish *SerRS*) and *Vegfa* in the zebrafish treated with MEQ or dimethyl sulfoxide (DMSO). Data are plotted as the mean ± SEM (*n* = 3; *P < 0.05; ***P < 0.001 using Student’s *t*-test). (D) Western blotting shows SerRS protein levels induced by MEQ in 4T1 cells. (E) Representative images of Matrigel plugs formed by 4T1 cells treated with MEQ or DMSO control. Scale bar represents 1 cm. (F–H) Immunofluorescence of CD31(F), immunochemical staining of SerRS (G) and VEGFA (H) in the Matrigel plugs and the quantification of positively-stained cells are plotted as the mean ± SEM (*n* = 5; *P < 0.05; ***P < 0.001 using Student’s *t*-test. Scale bars represent 100 μm.

To investigate the effect of MEQ on angiogenesis *in vivo*, we first utilized the transgenic zebrafish Tg [Fli1a: EGFP] with EGFP specifically expressed in vascular endothelial cells^20^. We determined that the safe dosage of MEQ that zebrafish could tolerate was below 2.5 μM (**Supplementary Figure S1**). As expected, when treated with 2.5 μM MEQ, more zebrafish showed hypo-intersegmental vessel (Hypo-ISV) branches than those treated with DMSO (*P* < 0.001) (**Figure 3A and 3B**). We observed increased SerRS expression (*P* < 0.05) and decreased Vegfa expression (*P* < 0.001) in MEQ-treated zebrafish (**Figure 3C**), suggesting that MEQ induced SerRS expression to negatively regulate vascular development in fish.

To further investigate if MEQ functioned as a tumor angiogenesis inhibitor in mammals, we next performed a Matrigel plug angiogenesis assay in mice^21^. The 4T1 cells, mouse breast cancer cells responsive to MEQ (**Figure 3D**), were mixed with 10 μM of MEQ and embedded in Matrigel before being injected subcutaneously into Balb/c mice. At 1 week post-inoculation, the Matrigel plugs containing tumor cells were dissected for angiogenesis examination (**Figure 3E**). We observed greatly inhibited blood vessel formation in the MEQ-treated group compared to the control group (*P* < 0.001) (**Figure 3E and 3F**). IHC staining showed that MEQ significantly increased SerRS expression and reduced VEGFA expression in the cells of the Matrigel plugs (*P* < 0.001) (**Figure 3G and 3H**). Taken together, these results strongly suggested that MEQ robustly suppressed angiogenesis *in vivo* by enhancing SerRS expression.

### MEQ suppressed the progression of breast cancer in a mouse allograft tumor model

**Figure 4:**
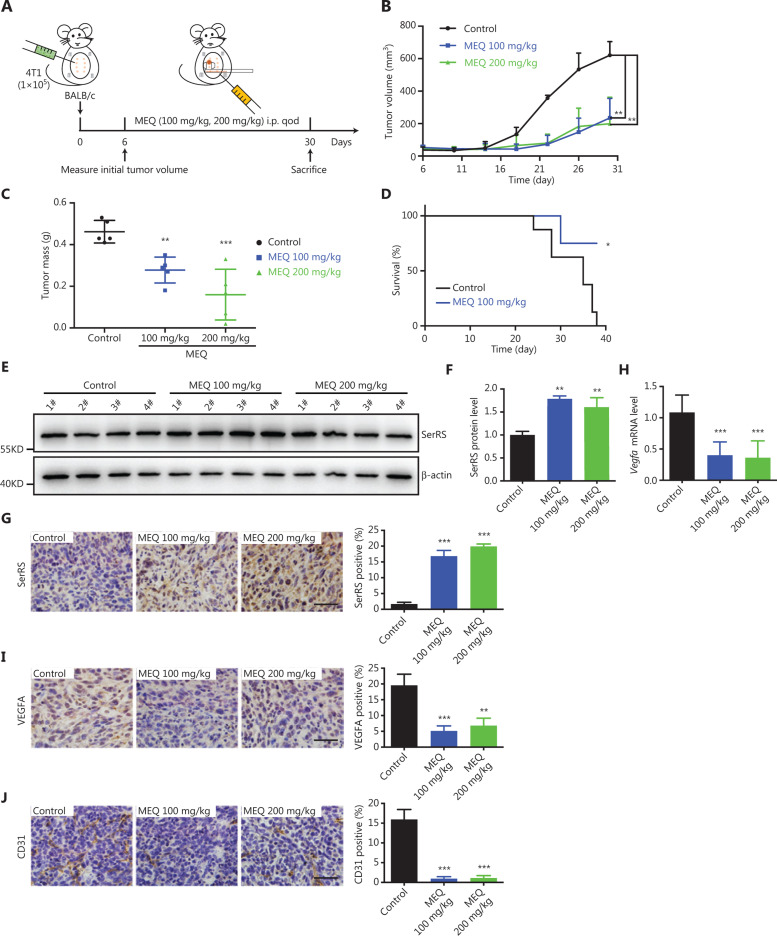
MEQ suppressed the progression of breast cancer allografts in mice. (A) Scheme of mouse experiments based on the breast cancer allograft model. qod: quaque omni die. (B) Tumor growth curve. (*n* = 5, **P < 0.01 using the Student’s* t*-test.). (C) Tumor weights at 30 days post-inoculation. (*n* = 5, **P < 0.01; ***P < 0.001 using Student’s* t*-test). (D) Kaplan-Meier survival curves of tumor-bearing Balb/c mice treated with MEQ (100 mg/kg) or dimethyl sulfoxide control (*n* = 8, *P < 0.05 using the log-rank test). (E, F) Western blotting to show SerRS protein levels in tumor tissues (E) and the densitometry quantification. (F) Data are plotted as the mean ± SEM (*n* = 4; **P < 0.01; ***P < 0.001 using Student’s* t*-test). (G) Immunohistochemical staining of SerRS in tumor tissue and its quantification (*n* = 5; ***P < 0.001 using Student’s* t*-test). (H) The qRT-PCR shows VEGFA mRNA levels of tumor tissues. (I, J) Immunohistochemical staining of VEGFA (I) and CD31 (J) in tumor tissues and quantification of positive cell numbers plotted as the mean ± SEM (*n* = 5; **P < 0.01; ***P < 0.001 using Student’s* t*-test). All scale bars represent 100 μm.

To further evaluate the anti-tumor effects of MEQ *in vivo*, we used a breast cancer allograft mouse model. The 4T1 highly malignant murine breast cancer cells were subcutaneously inoculated into female Balb/c mice. Six days after inoculation, the mice were treated with MEQ (100 mg/kg and 200 mg/kg) or DMSO every 2 days (**Figure 4A**). We observed greatly inhibited tumor growth in MEQ-treated mice (**Figure 4B and 4C**) and prolonged survival of tumor-bearing mice upon MEQ treatment (*P* < 0.05) (**Figure 4D**). MEQ-enhanced SerRS expression was also observed in tumor tissues by western blotting and IHC staining (**Figure 4E–4G**). VEGFA expression was also reduced by MEQ in tumor tissues (**Figure 4H and 4I**). As a result, dramatically inhibited tumor angiogenesis shown by IHC staining for CD31 was also observed in MEQ-treated mice (*P* < 0.001) (**Figure 4J**). Accordingly, poor tumor vascularization inhibited cell proliferation (**Supplementary Figure 2A**) and triggered more cell apoptosis (**Supplementary Figure 2B**).

To exclude the possibility that MEQ-inhibited tumor progression was due to its high toxicity in mice, we measured the weight change of BALB/c mice during MEQ treatment and found no statistical difference when compared with the control groups (**Supplementary Figure S3A**). We also performed hemogram tests for mice treated with MEQ (**Supplementary Table S1**), and found that every index was within the normal range and comparable to the control group. Furthermore, we examined H&E-stained sections of major organs including the heart, liver, spleen, lung, kidney, and brain, and found no pathological damage to the organs of MEQ-treated mice (**Supplementary Figure S3B**). Together, these results suggested a low toxicity of MEQ. Furthermore, its inhibitory role on tumor progression was due to regulation of the SerRS-VEGFA pathway.

### MEQ inhibited the growth of human TNBC xenografts in mice

**Figure 5:**
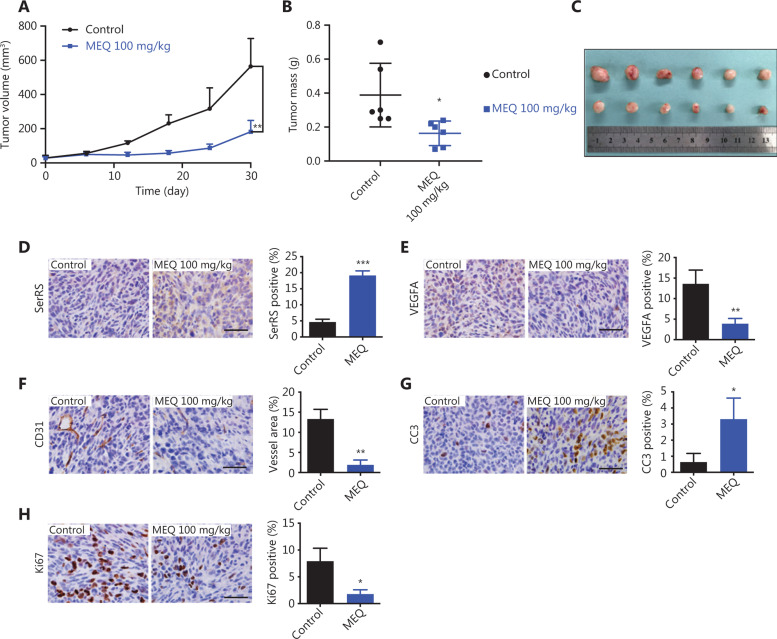
MEQ inhibits the growth of human triple-negative breast cancer (TNBC) xenografts in mice. (A) The growth curves of MDA-MB-231 xenografts in mice treated with MEQ or the dimethyl sulfoxide control. Data are plotted as the mean ± SD. (B, C) At 30 days post-inoculation, the xenografts were dissected and weighted, *n* = 5. D-H Immunohistochemical staining for SerRS (D), VEGFA (E), CD31 (F), CC3 (G), and Ki67 (H) in the xenografts; *n* = 5. All scale bars represent 100 μm and all bar graphs are plotted as the mean ± SEM (ns, not significant; *P < 0.05; **P < 0.01; ***P < 0.001 using Student’s* t*-test).

The MDA-MB-231 cell line-derived xenograft (CDX) mouse model can represent TNBC characteristics and is frequently used in Phase II clinical trials to test the efficacy of anti-cancer drugs^22,23^. Thus, we further examined the effect of MEQ on human TNBC xenografts in immunodeficient NOD-SCID mice. Consistently, we observed that MEQ strongly suppressed the growth of MDA-MB-231 xenografts (**Figure 5A–5C**). In addition, MEQ treatment also resulted in upregulated SerRS (*P* < 0.001) and downregulated VEGFA (*P* < 0.01) in TNBC xenografts (**Figure 5D and 5E**). Accordingly, CD31 staining showed dramatically inhibited angiogenesis in MEQ-treated xenografts (*P* < 0.01) (**Figure 5F**), leading to increased tumor cell apoptosis and inhibited cell proliferation as measured by IHC staining of CC3 (*P* < 0.05) and Ki-67 (*P* < 0.05), respectively (**Figure 5G and 5H**).

### MEQ activated SerRS expression by targeting MTA2

**Figure 6:**
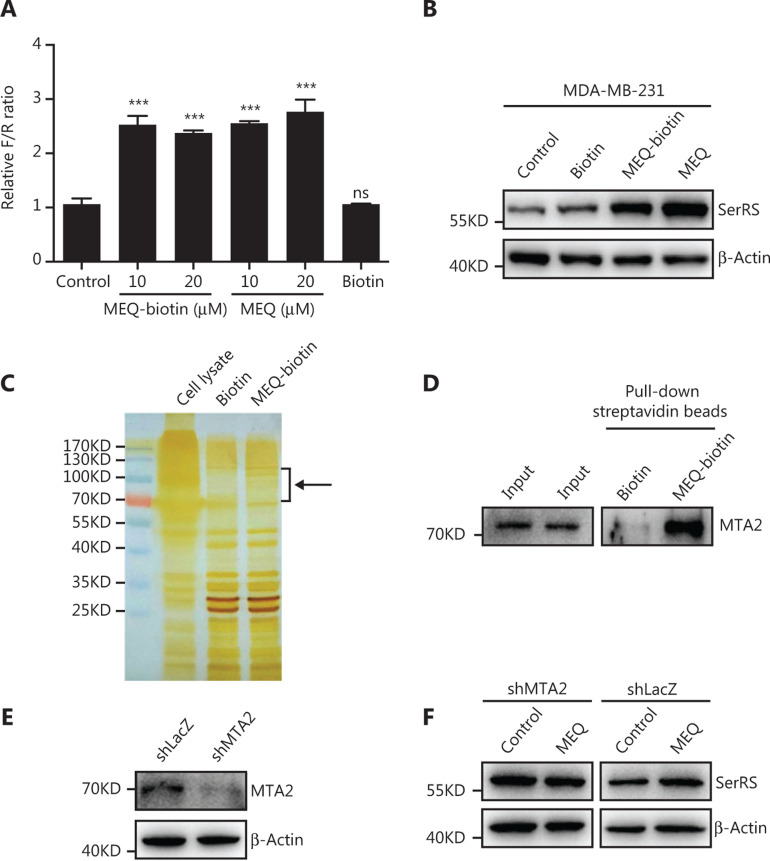
MEQ targets MTA2 to regulate SerRS expression. (A) Dual-luciferase reporter assay for biotin-conjugated MEQ (MEQ-biotin). Values are the mean ± SEM. ns, not significant, ***P < 0.001 using analysis of variance. (B) Western blots show that MEQ-biotin is as active as MEQ in regulating SerRS expression. (C) MEQ-interacting proteins were pulled down from the MDA-MB-231 cell lysate with streptavidin beads and analyzed by SDS-PAGE and silver staining. The black arrow indicates protein bands that may specifically interact with MEQ. (D) Western blotting to confirm that MTA2 was co-purified with MEQ-biotin. (E) Western blotting to show the knockdown efficiency of shRNA against MTA2 (shMTA2) in MDA-MB-231 cells. (F) Western blotting to show the protein levels of MEQ required to induce SerRS expression in MTA2-silenced (shMTA2) or control (shLacZ) MDA-MB-231 cells.

To further investigate the direct target of MEQ, through which MEQ enhances SerRS transcription, we utilized a streptavidin-biotin affinity pull-down assay. First, we linked biotin to the nitrogen of MEQ (**Supplementary Figures S4–S6**), which did not affect its function in activating SerRS expression (**Figure 6A and 6B**). Next, we incubated the biotin-labelled MEQ (MEQ-biotin) with MDA-MB-231 cell lysates and affinity purified the complex using streptavidin-conjugated beads. Following SDS-PAGE and silver staining, we observed a few specific protein bands between 70 kDa and 120 kDa in MEQ-biotin purified elutes compared with that in biotin purified elutes (**Figure 6C**). Mass spectrometric analysis of these specific bands suggested that MTA2, a cofactor in the NuRD transcriptional regulatory complex, was a candidate target of MEQ. We further confirmed the interaction between MTA2 and MEQ by western blot analysis on biotin-MEQ pull-down samples (**Figure 6D**). To further investigate the involvement of MTA2 in MEQ-activated SerRS expression, we knocked down MTA2 by shRNA (**Figure 6E**). When MTA2 was silenced, MEQ could no longer upregulate SerRS expression in MDA-MB-231 cells (**Figure 6F**), strongly suggesting that MTA2 was a key mediator of MEQ, which induced SerRS expression and subsequently inhibited tumor angiogenesis.

**Figure 7:**
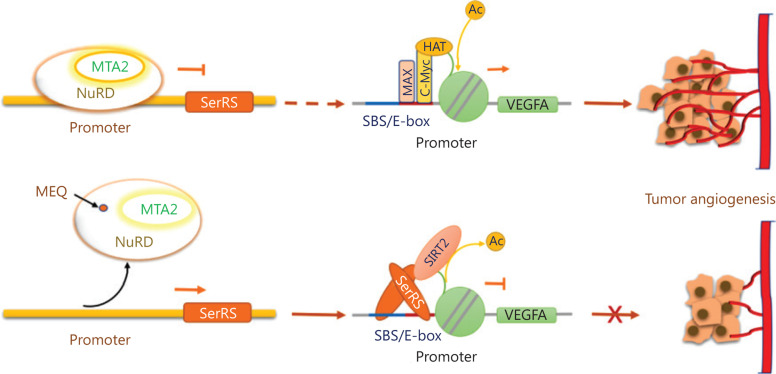
Mechanistic model to show how MEQ inhibits triple-negative breast cancer (TNBC) angiogenesis by regulating the MTA2/SerRS/VEGFA axis. The MTA2-containing NuRD deacetylase complex binds to the *SerRS* promoter to suppress its transcription, which allows more SerRS competitor, i.e., c-Myc proteins, to bind to the *VEGFA* promoter and activate VEGFA expression and tumor angiogenesis. MEQ interacts with MTA2 and may cause the release of the NuRD complex from the SerRS promoter, resulting in elevated expression of SerRS, which competes off the c-Myc and binds to the VEGFA promoter to suppress its transcription and inhibit tumor angiogenesis.

Taken together, these results suggested that MEQ interacted with MTA2, which is a nonenzymatic subunit in the nucleosome remodeling and histone deacetylase (NuRD) complex, to upregulate SerRS expression, and inhibit the expression of VEGFA and TNBC angiogenesis (**Figure 7**).

## Discussion

Angiogenesis plays an important role in the growth and progression of TNBC. Several groups have reported that VEGFA is expressed at significantly higher levels in TNBC patient tumors compared to those of non-TNBC patients^24–26^. Thus, drugs targeting the VEGFA pathway could be an effective treatment for TNBC. However, VEGFA-targeted drugs, such as the bevacizumab neutralizing antibody, have only shown a slight benefit for TNBC patients at the cost of adverse side effects^10,11^. Other small inhibitors targeting receptor tyrosine kinase (RTK) have relatively low general response rates in conjunction with particular toxicity profiles^27^. Recent reports showed that drugs targeting VEGFA itself or its receptors have always resulted in drug resistance, primarily due to the activation of alternative proangiogenic mechanisms in the tumors^28–30^. Thus, targeting a transcriptional factor that regulates multiple factors and signaling pathways important for angiogenesis could be a better solution. We found that nuclear-localized SerRS was a robust transcriptional repressor of VEGFA, through direct competition with c-Myc and NFκB for binding to the VEGFA promoter^12,13^. In addition, our previous studies showed that SerRS triggered the senescence of cervical tumor cells in mice by shortening telomeres^31^. Because MEQ treatment can increase the expression of SerRS in MDA-MB-231 cells, this compound could be a potential and multifunctional drug against TNBC and potentially against other cancers.

MEQ is an isoflavone derivative that can act as a phytoestrogen in mammals^32^. There is controversy about whether isoflavones are risk factors or effective treatments for breast cancer^33–39^. Most studies have shown that the carcinogenic character of isoflavone is due to its binding to estrogen receptors (ER)^40,41^. Because TNBC is ER negative, it might be one of the reasons why MEQ is effective in our TNBC models. Consistent with the anti-angiogenesis and anti-tumor functions of MEQ to TNBC, other researchers have reported that isoflavones have anti-proliferative properties in TNBC, both *in vitro* and *in vivo*^40,42–44^. Specifically, isoflavones can decrease microvascular density, reduce circulating levels of VEGF, and increase endostatin levels in rats with mammary tumors^45^. Thus, our finding that MEQ inhibited the expression of VEGFA in TNBC through the MTA2/SerRS/VEGFA pathway revealed the underlying mechanism for isoflavones and their derivatives, allowing the optimization of this natural molecule for cancer treatment.

Isoflavones, like formononetin, has increased benefits with fewer side effects when used as a combination drug with chemotherapy or as a postoperative adjuvant therapy^46,47^. We also found that MEQ displayed few adverse effects in mice, suggesting that MEQ may have clinical applications in combination with other chemotherapeutic drugs in TNBC treatment. Besides the anti-angiogenesis function of MEQ, we also found that MEQ inhibited TNBC proliferation and promoted apoptosis *in vivo*, similar to other isoflavones and their derivatives^40,42–44^.

The NuRD complex has been found to interact with different factors to promote cancer development^48–50^. The potential target of MEQ, MTA2, as one nonenzymatic subunit in the NuRD complex, confers functional specificity for the NuRD complex by associating with different transcription factors or targeting distinct gene loci^50^. Moreover, MTA2 is found to be overexpressed in human breast cancer^51^, and the VEGF signaling pathway was able to be modulated by targeting MTA2 expression in non small cell lung cancer cells^52^. Thus, characterizing drugs that target NuRD subunits for cancer therapy should be more specific and effective than those targeting pan-epigenetic modification enzymes. Our study is the first to show the connection between MTA2 and SerRS in modulating VEGFA expression in MDA-MB-231 cells. Our finding that MTA2 could be targeted by MEQ also provides a way to manipulate MTA2-mediated gene expression, which could be developed for cancer treatment. Further studies are needed to clarify the exact mechanism of MEQ in regulating MTA2 function.

In conclusion, our study identified an anti-angiogenesis compound, MEQ, which inhibited the progression of TNBC by targeting the MTA2/SerRS/VEGFA pathway.
