# Supplementary material for: An isoflavone derivative potently inhibits the angiogenesis and progression of triple-negative breast cancer by targeting the MTA2/SerRS/VEGFA pathway
Source: Cancer Biol Med. 2020 Aug 15;17(3):693–706. doi: 10.20892/j.issn.2095-3941.2020.0010 (PMC7476100; doi:10.20892/j.issn.2095-3941.2020.0010)
Supplement: Supplementary file 1 [file cbm-17-693-s001.pdf]

Supplementary materials

Table S1 Hemogram values of Balb/c mice with/without MEQ

Parameters	Control	MEQ (200 mg/kg)
WBC	5.94 ± 1.53	5.69 ± 0.68
Neutrophil granulocyte %	3.95 ± 1.86	4.05 ± 2
Lymphocyte %	95.43 ± 2.35	95.4 ± 2.17
Monocyte %	0.04 ± 0.06	0.04 ± 0.08
Eosinophilic granulocyte %	0.61 ± 0.51	0.5 ± 0.41
Basophilic granulocyte %	0 ± 0	0.02 ± 0.03
Neutrophil %	0.22 ± 0.04	0.24 ± 0.13
LY	5.69 ± 1.59	5.42 ± 0.56
MO	0 ± 0.01	0 ± 0.01
EO	0.03 ± 0.02	0.03 ± 0.03
BA	0 ± 0	0 ± 0
RBC	10.6 ± 0.36	10.53 ± 0.23
Hemoglobin	154.33 ± 2.08	146.67 ± 2.89
Hematocrit	35.27 ± 0.86	34.3 ± 0.46
MCV	33.4 ± 0.61	32.5 ± 0.8
MCH	14.57 ± 0.4	13.9 ± 0
MCHC	438 ± 6.08	428 ± 11
RDW	13.43 ± 0.25	13.87 ± 0.31
PLT	656.67 ± 51.6	641 ± 110.69
PDW	12.3 ± 0.46	12.17 ± 0.42
MPV	4.77 ± 0.25	4.47 ± 0.21
Platelet	0.31 ± 0.04	0.29 ± 0.04

Hemogram parameters of mice treated with MEQ or the dimethyl sulfoxide control. All values are presented as mean ± SD (n = 3 in each group). WBC, white blood cell; LY, lymphocyte; MO, mononuclear cell; EO, eosnophils; BA, basophil granulocyte; RBC, red blood cell; MCV, mean corpuscular volume; MCH, mean corpuscular hemoglobin; MCHC, mean corpuscular hemoglobin concentration; RDW, red blood cell distribution width; PLT, blood platelet; PDW, platelet distribution width; MPV, mean platelet volume.

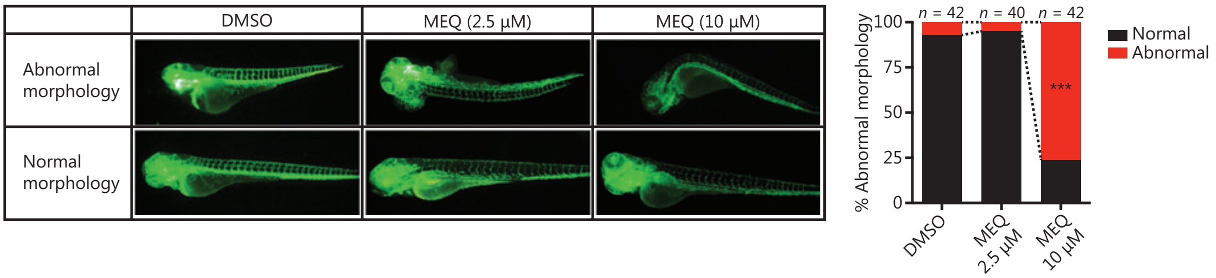

Figure S1 The safe dosage range of MEQ was tested in zebrafish. The fertilized eggs of Tg (Fli1a: GFP) zebrafish at the 1–2 cell stage were injected with indicated dosages of MEQ and the morphology of the fish at 72 h post-fertilization was photographed. The percentages of fish with normal and abnormal morphologies were plotted as a bar graph (χ^2-test, ***$P < 0.001$). Scale bar: 0.2 mm.

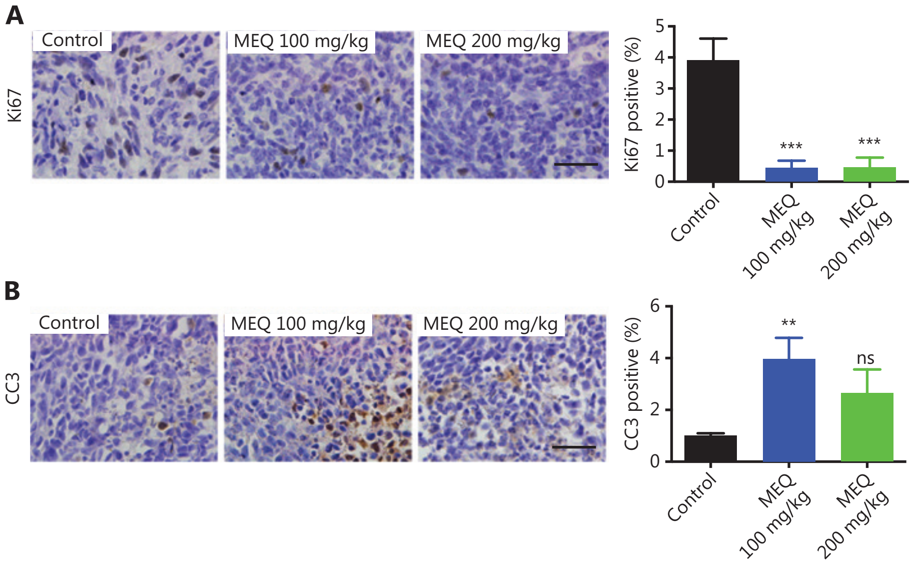

Figure S2 Immunohistochemical staining for Ki67 (A) and CC3 (B) in 4T1 allografts dissected from mice and the quantification of positively stained cells. Data are plotted as the mean ± SEM ($n = 5$; **$P < 0.01$; ***$P < 0.001$ using Student's t-test). Scale bars: 100 μm.

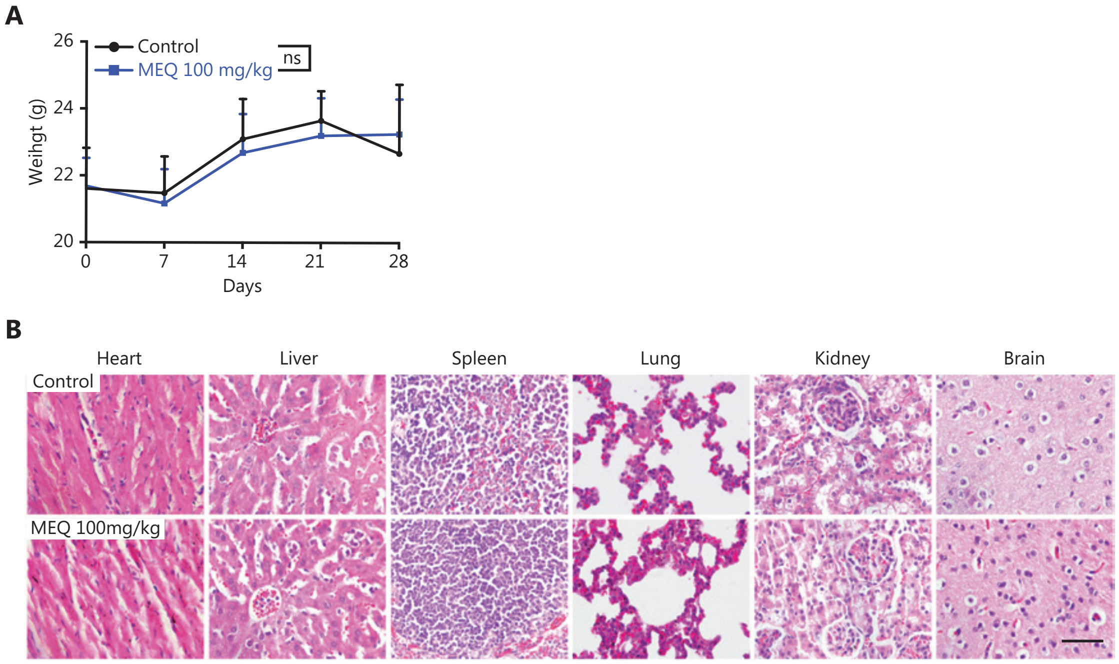

Figure S3 The MEQ toxicity tests in mice. (A) Hematoxylin and eosin staining for the sections of hearts, livers, spleens, lungs, kidneys, and brains of mice treated with 100 mg/kg MEQ or the dimethyl sulfoxide (DMSO) control. Scale bar represents 100 μm. (B) Weight changes of mice treated with DMSO or MEQ (100 mg/kg, intraperitoneal injections, once a day) for 4 weeks.

Scheme of the synthesis of Biotin-TEG-N3

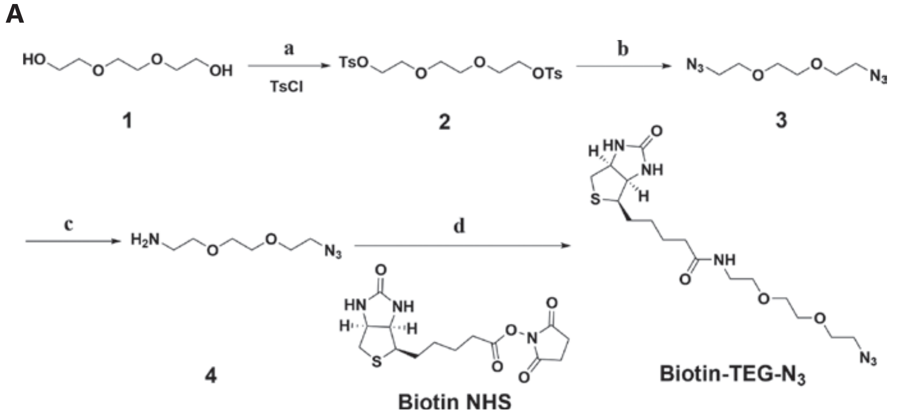

Scheme of the synthesis of MEQ-TEG-Biotin

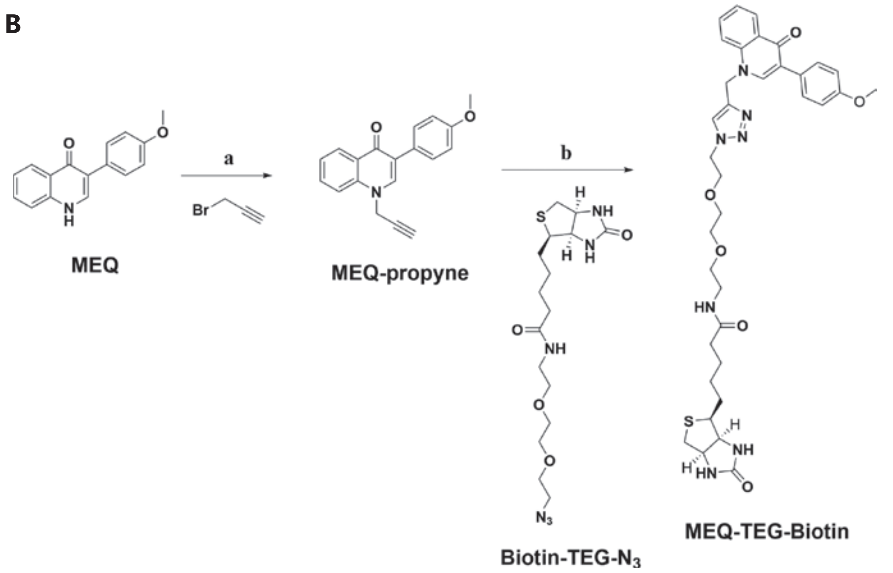

Figure S4 Synthesis of MEQ-biotin. (A) Synthesis of biotin-TEG-N_3. Reagents and conditions: (a) KOH, $CHCl_3$, 0 °C, 1.5 h; (b) NaN_3, DMF, 65 °C, 24 h; (c) PPh_3, EtOAc/HCl/H_2O, room temperature, 12 h; (d) DIEA, DMF/H_2O, 35 °C, 12 h. (B) Synthesis of MEQ-TEG_3-biotin. Reagents and conditions: (a) K_2CO_3, DMF, 0 °C, 8 h; (b) Et_3N, $CuSO_4$, Buffer, 35 °C, 5 h.

A

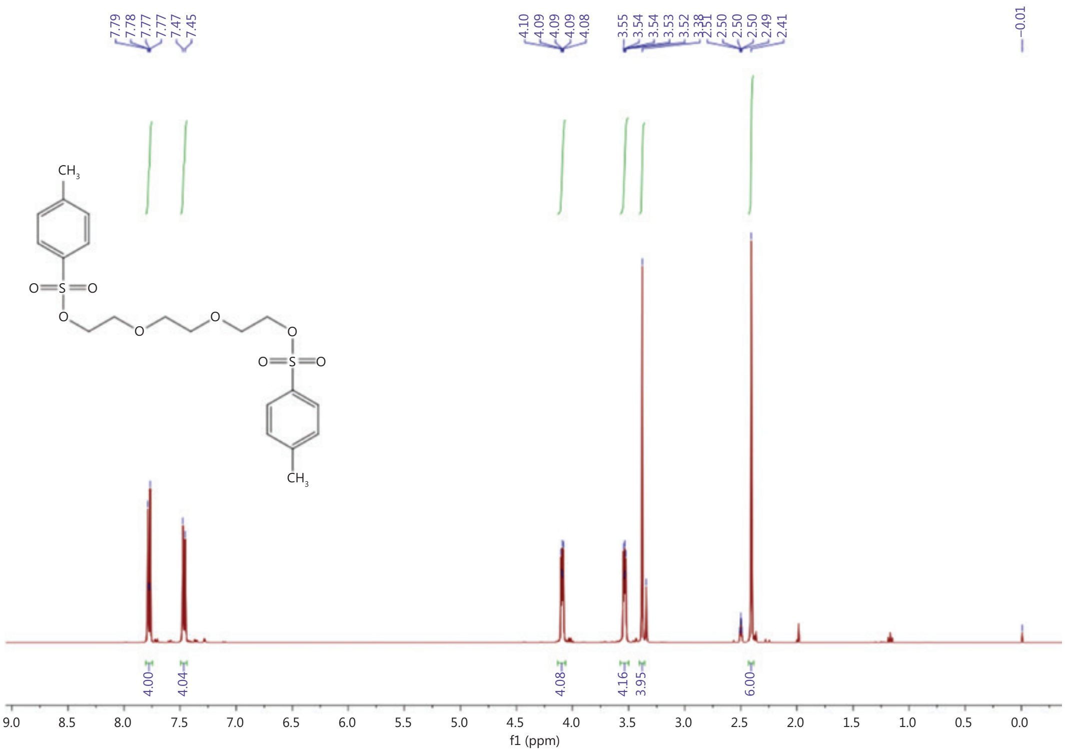

B

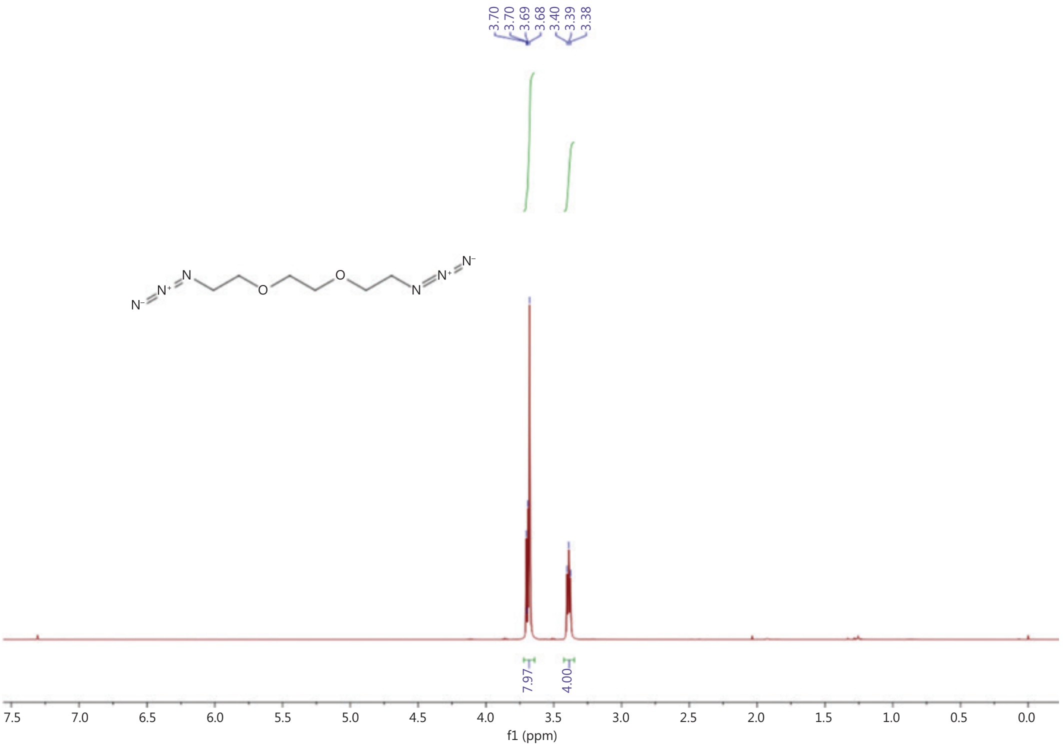

Figure S5 (continued)

C

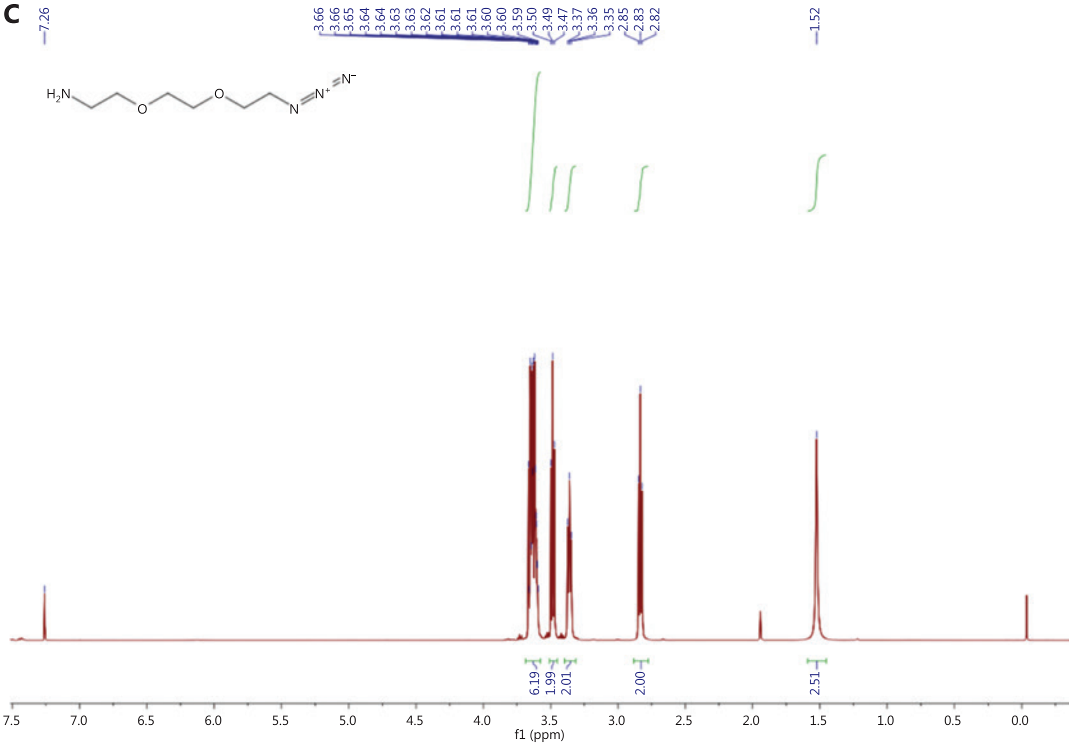
7.26
3.66 3.66 3.65 3.64 3.64 3.63 3.63 3.62 3.61 3.61 3.61 3.60 3.60 3.59 3.50 3.49 3.47 3.37 3.36 3.35 2.85 2.83 2.82
1.52
H2N O O N N+ N−
6.19 1.99 2.01 2.00 2.51
7.5 7.0 6.5 6.0 5.5 5.0 4.5 4.0 3.5 3.0 2.5 2.0 1.5 1.0 0.5 0.0
f1 (ppm)

D

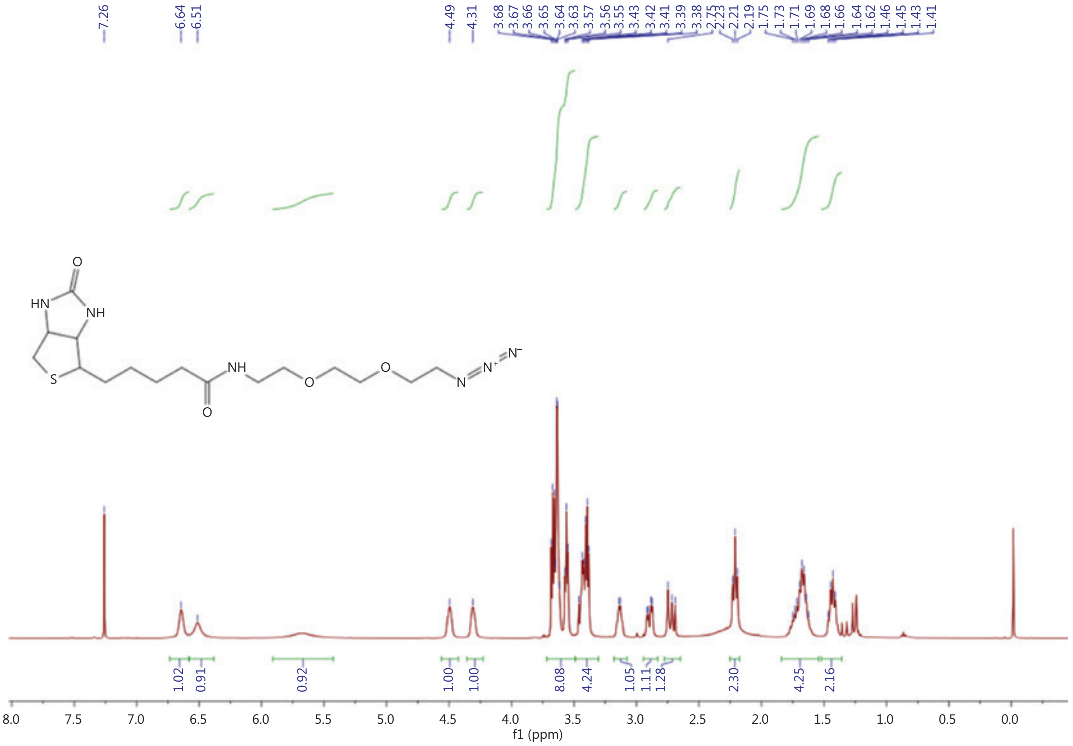
7.26
6.64 6.51
4.49 4.31
3.68 3.67 3.66 3.65 3.64 3.63 3.57 3.56 3.55 3.43 3.42 3.41 3.39 3.38 2.75 2.23 2.21 2.19 1.75 1.73 1.71 1.69 1.68 1.66 1.64 1.62 1.46 1.45 1.43 1.41
O HN NH S NH O O O N N+ N−
1.02 0.91 0.92 1.00 1.00 8.08 4.24 1.05 1.11 1.28 2.30 4.25 2.16
8.0 7.5 7.0 6.5 6.0 5.5 5.0 4.5 4.0 3.5 3.0 2.5 2.0 1.5 1.0 0.5 0.0
f1 (ppm)

Figure S5 (A) 1H NMR of TEG-diOTs.1H NMR (400 MHz, DMSO-d6) δ 7.81–7.75 (m, 4H), 7.46 (d, *J* = 8.0 Hz, 4H), 4.13–4.06 (m, 4H), 3.58–3.50 (m, 4H), 3.38 (s, 4H), 2.41 (s, 6H). Exact mass calculated for $C_{20}H_{26}O_8S_2$ [M+H]+: 459.1147; found 459.1150. (B) 1H NMR of TEG-diN_3. 1H NMR (400 MHz, CDCl3) δ 3.72–3.64 (m, 8H), 3.42–3.35 (m, 4H). Exact mass calculated for $C_6H_{12}N_6O_2$ [M+H]+: 201.1100; found 201.1102. (C) 1H NMR of N_3-TEG-NH_2. 1H NMR (400 MHz, $CDCl_3$) δ 3.69–3.57 (m, 6H), 3.49 (t, *J* = 5.2 Hz, 2H), 3.40–3.31 (m, 2H), 2.83 (t, *J* = 5.2 Hz, 2H), 1.52 (s, 2H). Exact mass calculated for $C_6H_{14}N_4O_2$ [M+H]+: 175.1195; found 175.1193. (D) 1H NMR of Biotin-TEG-N_3. 1H NMR (400 MHz, $CDCl_3$) δ 6.64 (s, 1H), 6.51 (s, 1H), 5.91–5.43 (m, 1H), 4.49 (s, 1H), 4.31 (s, 1H), 3.72–3.49 (m, 8H), 3.42 (dt, *J* = 9.9, 7.9 Hz, 4H), 3.13 (d, *J* = 4.3 Hz, 1H), 2.89 (dd, *J* = 12.8, 4.5 Hz, 1H), 2.78–2.65 (m, 1H), 2.21 (t, *J* = 7.3 Hz, 2H), 1.69 (ddt, *J* = 21.0, 13.7, 7.0 Hz, 4H), 1.44 (dd, *J* = 14.6, 7.3 Hz, 2H). Exact mass calculated for $C_{16}H_{28}N_6O_4S$ [M+H]+: 401.1971; found 401.1970.

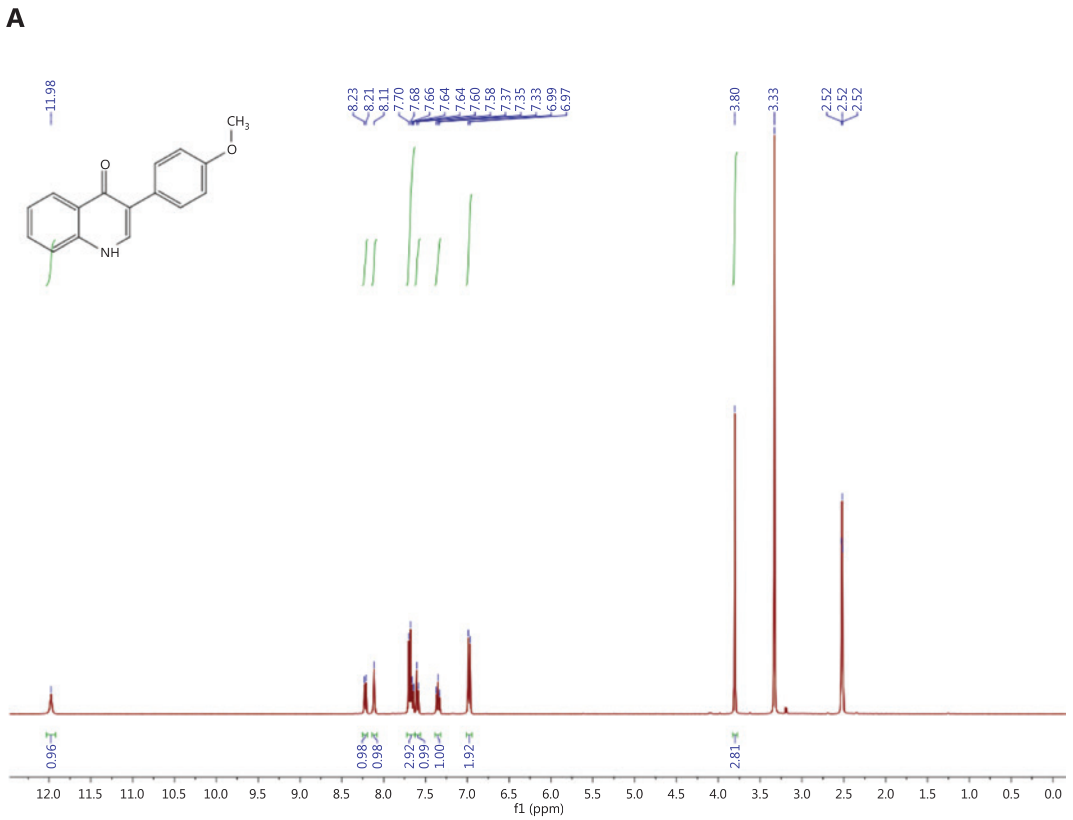

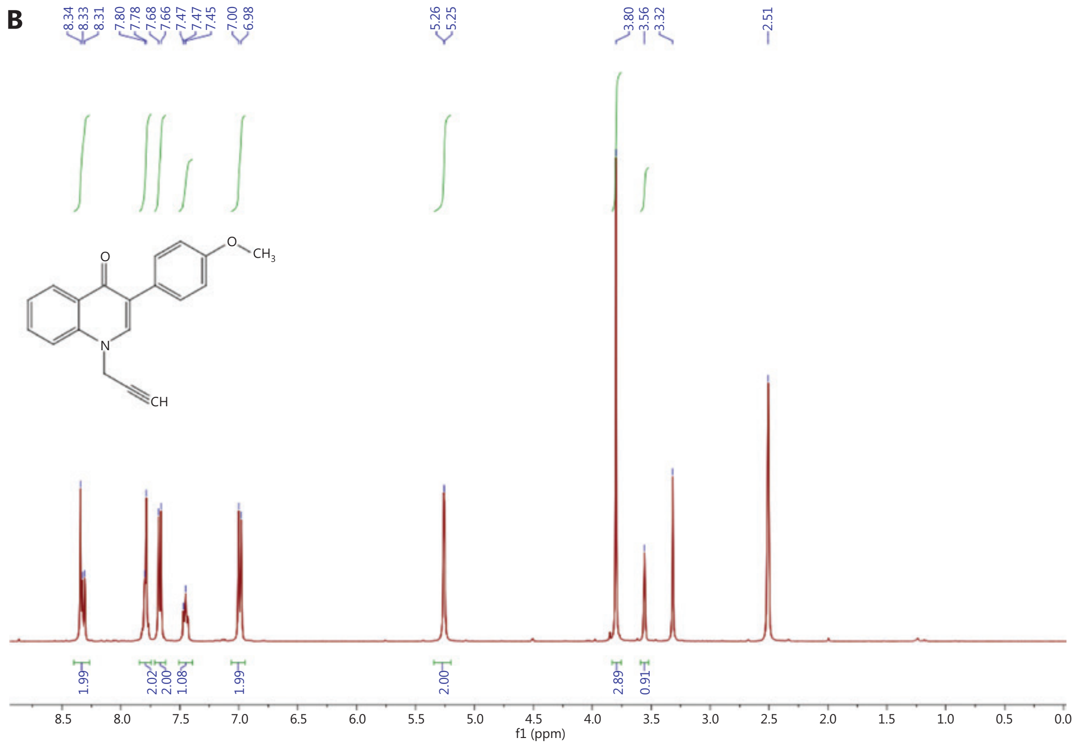

Figure S6 (continued)

C

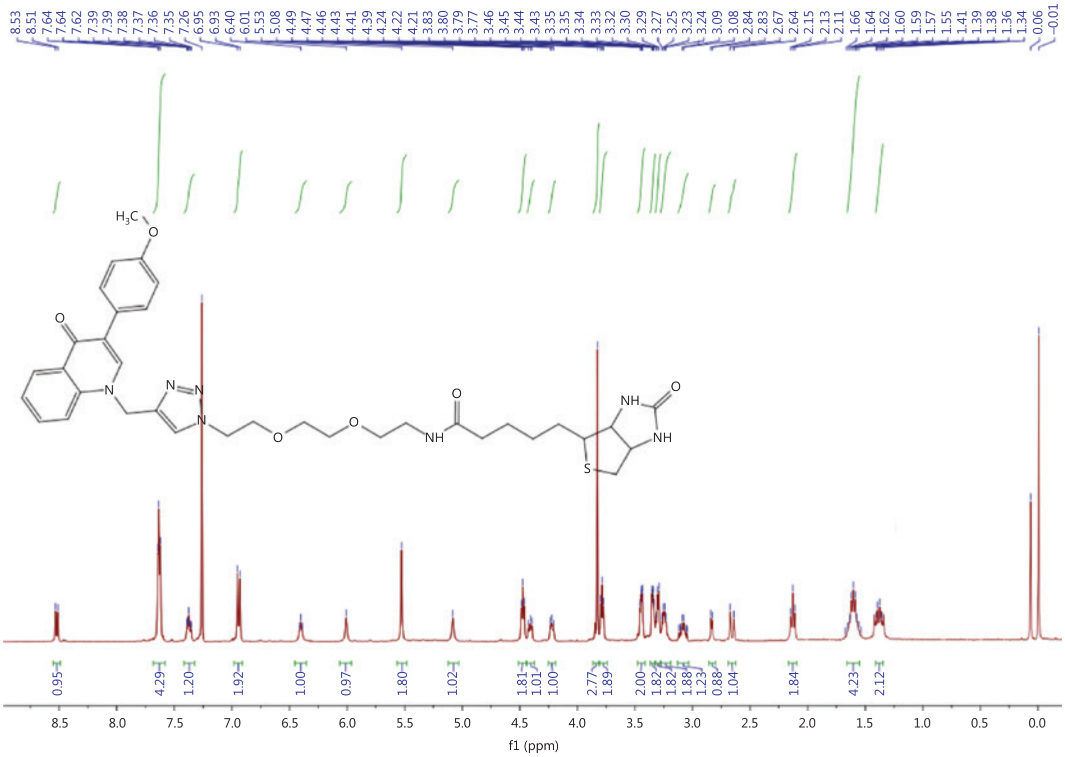

Figure S6 (A) ^{1}H NMR of MEQ. ^{1}H NMR (400 MHz, DMSO-d_6) δ 11.98 (s, 1H), 8.22 (d, J = 8.1 Hz, 1H), 8.11 (s, 1H), 7.72–7.63 (m, 3H), 7.59 (d, J = 8.2 Hz, 1H), 7.35 (t, J = 7.4 Hz, 1H), 6.98 (d, J = 8.7 Hz, 2H), 3.80 (s, 3H). Exact mass calculated for $C_{16}H_{13}NO_2$ [M+H]+: 252.1025; found 252.1026. (B) ^{1}HNMR of MEQ-propyne. ^{1}H NMR (400 MHz, DMSO-d_6) δ 8.40–8.26 (m, 2H), 7.79 (d, J = 5.1 Hz, 2H), 7.67 (d, J = 8.6 Hz, 2H), 7.51–7.39 (m, 1H), 6.99 (d, J = 8.6 Hz, 2H), 5.26 (d, J = 1.9 Hz, 2H), 3.80 (s, 3H), 3.56 (s, 1H). Exact mass calculated for $C_{19}H_{15}NO_2$ [M+H]+: 290.1181; found 290.1180. (C) ^{1}H NMR of MEQ-TEG-Biotin. ^{1}H NMR (400 MHz, $CDCl_3$) δ 8.52 (d, J = 7.9 Hz, 1H), 7.68–7.58 (m, 4H), 7.42–7.33 (m, 1H), 6.94 (d, J = 8.7 Hz, 2H), 6.40 (t, J = 5.2 Hz, 1H), 6.01 (s, 1H), 5.53 (s, 2H), 5.08 (s, 1H), 4.47 (t, J = 4.9 Hz, 2H), 4.44–4.37 (m, 1H), 4.25–4.19 (m, 1H), 3.83 (s, 3H), 3.78 (t, J = 4.9 Hz, 2H), 3.44 (dd, J = 5.4, 3.2 Hz, 2H), 3.34 (dd, J = 5.4, 3.1 Hz, 2H), 3.32–3.28 (m, 2H), 3.25 (dd, J = 9.3, 4.5 Hz, 2H), 3.13–3.03 (m, 1H), 2.83 (d, J = 4.8 Hz, 1H), 2.66 (d, J = 12.7 Hz, 1H), 2.13 (t, J = 7.3 Hz, 2H), 1.66–1.55 (m, 4H), 1.38 (dd, J = 13.7, 6.3 Hz, 2H). Exact mass calculated for $C_{35}H_{47}N_7O_6S$ [M+Na]+: 712.2893; found 712.2904.
